# Family Adaptation, Cohesion, and Social Climate in Parental Families of Women with Borderline Personality Disorder (BPD) and Mentally Healthy Women

**DOI:** 10.1192/j.eurpsy.2026.11909

**Published:** 2026-07-31

**Authors:** V. I. Rozhdestvenskiy, V. S. Lupashku

**Affiliations:** Department of Clinical Psychology, Saint Petersburg State Pediatric Medical University, Saint Petersburg, Russian Federation

## Abstract

**Introduction:**

The parental families of women with Borderline Personality Disorder **(**BPD) are characterized by a rigid family system structure, manifested in inflexible roles and rules, as well as compromised hierarchy and boundaries. These dysfunctions create conditions for the development of maladaptive coping strategies and contribute to impairments in self-regulation and interpersonal functioning, which are characteristic of them. The social family climate refers to the behavioral rules accepted within the family that influence relationships among its members. Family cohesion implies the degree of emotional bonding among family members, while family adaptation refers to the ability of a family system to be flexible and change its structure, roles, and rules in response to situational and developmental stress.

**Objectives:**

The aim of this study was to explore the relationships between the levels of family adaptation and cohesion, and indicators of the social family climate in the parental families of women with BPD compared to the control group of mentally healthy women.

**Methods:**

Data were collected in 2025 using an online form developed by the authors. The sample consisted of 20 women diagnosed with Borderline Personality Disorder **(**BPD) and 20 women in the control group of mentally healthy women, aged 18–25. The Family Adaptation and Cohesion Scales (FACES-III) were used to assess family adaptation and cohesion, and the Family Environment Scale (FES) was used to evaluate the social family climate. Both questionnaires were adapted for Russian-speaking respondents.

**Results:**

In the parental families of women with BPD, Family Adaptation was negatively correlated with the Control (r = -0.5, p < 0.05) and Achievement Orientation (r = -0.52, p < 0.05) subscales. Family Cohesion was positively correlated with the Moral-Religious Emphasis subscale (r = 0.54, p < 0.05). In the control group, Family Cohesion was positively associated with the Independence (r = 0.5, p < 0.05), Conflict (r = 0.52, p < 0.05), and Expressiveness (r = 0.48, p < 0.05) subscales.

**Conclusions:**

In the parental families of women with BPD, family adaptation is compromised by high levels of control and a strong achievement orientation. Furthermore, family cohesion appears formalistic, mediated by moral and religious pressure. This combination creates a traumatic environment that hinders the development of a stable self-identity and emotional regulation skills, leading to significant impairments in interpersonal relationships. In contrast, in the parental families of the control group, family cohesion is associated with flexibility, independence, and open expression of feelings, indicating a more adaptive and healthy family system.

**Disclosure of Interest:**

None Declared

